# Identification of Nitrogen-Fixing Genes and Gene Clusters from Metagenomic Library of Acid Mine Drainage

**DOI:** 10.1371/journal.pone.0087976

**Published:** 2014-02-03

**Authors:** Zhimin Dai, Xue Guo, Huaqun Yin, Yili Liang, Jing Cong, Xueduan Liu

**Affiliations:** 1 School of Minerals Processing and Bioengineering, Central South University, Changsha, P. R. China; 2 Key Laboratory of Biometallurgy of Ministry of Education, Central South University, Changsha, P. R. China; Technical University of Denmark, Denmark

## Abstract

Biological nitrogen fixation is an essential function of acid mine drainage (AMD) microbial communities. However, most acidophiles in AMD environments are uncultured microorganisms and little is known about the diversity of nitrogen-fixing genes and structure of *nif* gene cluster in AMD microbial communities. In this study, we used metagenomic sequencing to isolate *nif* genes in the AMD microbial community from Dexing Copper Mine, China. Meanwhile, a metagenome microarray containing 7,776 large-insertion fosmids was constructed to screen novel *nif* gene clusters. Metagenomic analyses revealed that 742 sequences were identified as *nif* genes including structural subunit genes *nifH*, *nifD*, *nifK* and various additional genes. The AMD community is massively dominated by the genus *Acidithiobacillus*. However, the phylogenetic diversity of nitrogen-fixing microorganisms is much higher than previously thought in the AMD community. Furthermore, a 32.5-kb genomic sequence harboring *nif*, *fix* and associated genes was screened by metagenome microarray. Comparative genome analysis indicated that most *nif* genes in this cluster are most similar to those of *Herbaspirillum seropedicae*, but the organization of the *nif* gene cluster had significant differences from *H. seropedicae*. Sequence analysis and reverse transcription PCR also suggested that distinct transcription units of *nif* genes exist in this gene cluster. *nifQ* gene falls into the same transcription unit with *fixABCX* genes, which have not been reported in other diazotrophs before. All of these results indicated that more novel diazotrophs survive in the AMD community.

## Introduction

Biological nitrogen fixation occurs in more than 100 genera distributed among several of the major phylogenetic divisions of *Bacteria* and *Archaea* [1]. Bacterial *nif* genes are known to encode the components of the nitrogenase enzyme complex. The structural subunit of dinitrogenase reductase and the 2 subunits of dinitrogenase are encoded by the *nifH*, *nifD*, and *nifK* genes, respectively. In many diazotrophs like *Azotobacter vinelandii* [2], *Herbaspirillum seropedicae* [3], *Pseudomonas stutzeri* [4], and *Bradyrhizobium japonicum* [5], these proteins have similar sequences and common structures and functions. Furthermore, genetic and biochemical analyses revealed that many additional *nif* genes, including *nifE*, *nifN*, *nifX*, *nifQ*, *nif W*, *nifV*, *nifA*, *nifB*, *nifZ*, and *nifS*, play roles in the regulation of *nif* genes and maturation processes of inactive products, such as electron transport and FeMo-cofactor biosynthesis and assembly [6], [7]. However, the linkage and arrangement of *nif* gene cluster vary considerably in many diazotrophs. The apparent conservation of gene arrangement suggests that they serve some important function, perhaps in regulation of nitrogen fixation[8]. In addition, the *fixABCX* genes first identified in *Rhizobium meliloti* [9] and subsequently in other diazotrophs were reported to encode a membrane complex participating in electron transfer to nitrogenase [10].

Acid mine drainage (AMD) is the outflow of acidic water from metal or coal mines, which causes worldwide environmental problems. Despite the extreme acidity, heat, and high concentrations of toxic metals, a wide variety of microorganisms populate AMD environments. These organisms can sustain by the oxidation of sulfide minerals, CO_2_, O_2_, and N_2_ derived from air, and phosphate liberated by water-rock interaction [11]. Since the input of externally-derived fixed nitrogen is negligible, biological nitrogen fixation is an important function of AMD microbial communities. However, a few microorganisms in AMD environments are represented by isolates that have been cultivated and described [12] and only several species in three genera (*Acidithiobacillus*, *Leptospirillum* and *Methylacidiphilum*) have been proved to have nitrogen-fixing ability until now [13], [14]. Little is known about the diversity and structure of nitrogen-fixing genes of AMD microbial community.

Metagenomic sequencing and metagenomic libraries recently provide powerful tools to isolate novel genes or gene clusters from unexploited gene pools in uncultured microorganisms [15]. In this study, we found evidences of novel *nif* genes present in acid mine drainage from Dexing Copper Mine, China, using metagenomic sequencing. We also present the phylogenetic analysis of *nif* genes in AMD community. To understand the organization of *nif* gene clusters of uncultured microorganisms in AMD community, a metagenome microarray was constructed to screen *nif*-containing fosmids. A comparative analysis of the nitrogen-fixing gene cluster in different species revealed the presence of novel nitrogen-fixing gene cluster in AMD community. These results confirmed that there are more novel diazotrophs surviving in AMD community.

## Materials and Methods

### Sample permits

The studied locations are in a state owned by Jiangxi Copper Corporation. All necessary permits were obtained for the described field studies from Jiangxi Copper Corporation. Furthermore, our study did not harm the environment and did not involve endangered or protected species.

### Site description and sample collection

The microbial community growing on the surface of effusion pool beside the mill tailings at Dexing Copper Mine, China, was sampled in August 2008. The mill tailings containing low grade chalcopyrite and pyrites is the largest tailing heap in China. A total of 50 L of the original water sample was obtained at 0 to 10 cm below the water surface. The original water sample was filtered through 0.22 µm pore size filter (Millipore) immediately, and the filters were stored at −80°C until DNA extraction.

### Bacterial strains and plasmids

EPI300-T1^R^ (Epicentre, Madison, WI) was used as the host strain for fosmid library cloning. *Escherichia coli* BL21 (Tiangen) was used as the host strain for *nif* gene cloning and expression. The plasmids pCC2FOS (Epicentre, Madison, WI) and pET-28a (+) (Novagen) were used as the cloning vector and expression vector, respectively.

### DNA extraction, metagenomic sequencing and metagenomic library construction

Total DNA was extracted using the liquid nitrogen grinding method [16] and finally suspended in MilliQ water. The DNA sample was further determined in 1% (w/v) agarose gels, and NanoDrop measurements gave a concentration of 350 ng/µl with A_260_/A_280_ of 1.90. A total of 5 µg of total DNA was pyrosequenced using Roche 454 GS FLX system (Majorbio, China). Since the length of generated reads were long enough to annotate (90% reads >400 base pairs), assembly of the raw sequences was not performed. The unassembled metagenomic dataset was subjected to further analysis. At the same time, a metagenomic library was constructed using total DNA and CopyControl™ Fosmid Library Production Kit (Epicentre, Madison, WI) according to the manufacture's protocol. The collection of the metagenomic library contained total of 7,776 large-insert fosmid clones.

### *NifK* gene amplification

*NifK* sequences were PCR amplified with self-designed universal *nifK* primers sxnif_K1 (5′-CCTGGATGACCGAAGACGC-3′) and sxnif_K2 (5′-GGTGCCGCCTTCATACAT-3′). Amplification was performed in 50 µl reaction mixtures containing 1 µl of DNA extracts, 1 µl each of 10 µM forward and reverse primers, 25 µl of universal Taq PCR Master Mix(Tiangen Biotech, China),22 µl of deionized water. The PCR conditions for amplification were as follows: 94°C for 4 min, then 32 cycles of 94°C for 30 s, 51°C for 30 s, and 72°C for 45 s, followed by a final extension at 72°C for 10 min. PCR products of *nifK* were visualized on 2% agarose gels in TAE buffer and purified directly with the QIAquick PCR purification kit (Qiagen, Germany).

### Metagenome microarray construction, hybridization, sequencing and assembly

To screen *nif*-containing fosmids, metagenome microarray was constructed using metagenomic library mentioned above. Each clone was incubated in a shaking incubator at 37°C and 170 r.p.m in the presence of chloramphenicol and an inducer (Epicentre). After overnight incubations, cells were harvested and the fosmid DNA was extracted using QIAprep spin miniprep kit (Qiagen, Germany) according to the manufacturer's protocol. The fosmid DNAs were stored in final concentration of 40 ng·µl^−1^. 10 µl fosmid DNA of each clone was transferred to a 384-well microplate, and diluted 1∶1 (V/V) in 40% dimethyl sulfoxide (Sigma, USA). Then, the fosmid DNA of each clone was arrayed on microarray with two replicates using Genemachines OmniGrid Accent microarrayer (Genomic Solutions, USA). In addition, the following controls were spotted to check by hybridization, printing, and data analysis: (i) environmental DNA as positive controls, (ii) negative controls with *E. coli* genomic DNA, and (iii) blanks. The microarrays were post-treated as described previously[17], and stored in room temperature.

Here, we chose PCR products of *nifK* genes as probe for microarray hybridization, since NifK is indispensable in all diazotrophs. They were labeled with the Bioprime DNA Labeling kit (Invitrogen, Carlsbad, CA) according to the manufacture's protocol. Then, the labeled DNA was purified using a QIAquick PCR purification kit (Qiagen, Germany), concentrated into crystallization in a SpeedVac, and finally resuspended in 20 µl MilliQ water. And labeled DNA was mixed with hybridization solution. The hybridization solution contained 20 µl of labeled DNA, 65 µl of formamide (50%,v/v), 19.5 µl of 20×SSC (1×SSC containing 150 mM NaCl and 15 mM trisodium citrate), 9.1 µl of Herring Sperm DNA(10 mg/mL)(Promega, Madison, WI), 3.9 µl of 10% sodium dodecyl sulfate (SDS), 1.1 µl of DTT(0.1 M) in a total volume of 130 µl. Finally, the mixed solution was incubated at 98°C for 3 min, and then kept at 65°C. Microarray hybridization was performed at 54°C using the HS 4800Pro Hybridization station (TECAN, Switzerland). After hybridization, the microarray was visualized using a GenePix 4100A Microarray Scanner (Axon, USA). The normalized intensity of each spot was calculated as described previously[18], and positive clones in metagenomic library can be obtained based on the positive spots in metagenome microarray. Each positive clone was tested using PCR amplification with universal *nifK* primers mentioned above.

The verified positive fosmids were individually isolated using QIAprep spin miniprep kit (Qiagen), and pyrosequenced using Roche 454 GS FLX system (Majorbio, China). A total of 5 µg of fosmid DNA from each clone was tagged individually using a multiplex identifier adaptor containing a unique 10 base pair sequence that is recognized by the sequencing analysis software. Finally, each fosmid had ∼1.2 Mb of sequencing data for assembly, equivalent to the >30X clone coverage. And the average length of reads was 410 bp. Each of the fosmids was assembled into one single contig with the program Newbler [19].

### Annotation and analysis of genome fragments

Metagenomic sequencing yielded a total of 640,892 reads and 300 Mb of raw sequence. The individual metagenomic sequences >400 bp (approximately 90% sequences) were annotated using the non-redundant (NR) database, KEGG database, COG database. The sequences annotated as *nif* genes were selected to further analyze (Table.1). Protein-coding genes of fosmids were predicted using GLIMMER [20] and the RAST server [21], and further curated (Table.2). Identified ORFs were compared to known proteins in the non-redundant (NR) database, KEGG database and COG database using BLASTX [22], and all hits with e-value >1e-5 were considered nonsignificant. Other unassigned ORFs were annotated using the hmmpfam program of the HMMER package [23]. The hidden Markov models for the protein domains were obtained from the Pfam database 26.0 (http://pfam.sanger.ac.uk/). For comparative analysis, BLASTN and BLASTX searches among fosmids and different bacterial genomes were performed, leading to the identification of regions of similarity. To allow for the interactive visualization of genomic fragment comparisons, we used Artemis Comparison Tool [24]. Phylogenetic trees were constructed by the use of Molecular Evolutionary Genetics Analysis 4.0 (MEGA 4.0) software[25].

**Table 1:** detection of *nif* genes in metagenomic sequencing of acid mine drainage.

Gene	Function	sequence number
*nifH*	nitrogenase reductase	82
*nifD*	nitrogenase molybdenum-iron protein subunit alpha	127
*nifK*	nitrogenase molybdenum-iron protein subunit beta	93
*nifE*	nitrogenase molybdenum-cofactor biosynthesis protein	85
*nifN*	nitrogenase molybdenum-cofactor biosynthesis protein	75
*nifX*	iron-molybdenum cofactor processing protein	26
*nifA*	Nif-specific regulatory protein	89
*nifB*	FeMo cofactor biosynthesis protein	78
*nifQ*	molybdenum ion binding protein	1
*nifS*	cysteine desulfurase	11
*nifT*	nitrogen fixation protein	0
*nifU*	Fe-S cluster assembly protein	2
*nifV*	homocitrate synthase	62
*nifW*	nitrogenase stabilizing/protective protein	2
*nifZ*	iron-sulfur cofactor synthesis protein	1
*nifJ*	pyruvate-flavodoxin oxidoreductase	6
*nifL*	nitrogen fixation negative regulator	1
*nifP*	serine acetyltransferase	1
total		742

**Table 2:** List of ORFs from fosmid DX-1A-14, gene length, and similar genes in GenBank.

ORF	Gene length (bp)	Close relative (protein, [organism], identity^a^)
1	594	Nitrogenase MoFe protein [*Herbaspirillum seropedicae* SmR1] 80%
2	1515	MoFe cofactor biosynthesis protein NifE [*Burkholderia vietnamiensis* G4] 79%
3	1362	MoFe cofactor biosynthesis protein NifN [*Herbaspirillum seropedicae* SmR1] 68%
4	405	MoFe cofactor biosynthesis protein NifX [*Herbaspirillum seropedicae* SmR1] 74%
5	462	Hypothetical protein [*Herbaspirillum seropedicae* SmR1] 71%
6	204	Hypothetical protein [*Beijerinckia indica subsp. indica* ATCC 9039] 58%
7	315	4Fe-4S ferredoxin [*Rubrivivax benzoatilyticus* JA2] 70%
8	576	NifQ family protein [*Candidatus Accumulibacter phosphatis clade* IIA] 52%
9	294	Ferredoxin protein [*Herbaspirillum seropedicae* SmR1] 74%
10	1299	Ferredoxin protein [*Herbaspirillum seropedicae* SmR1] 79%
11	1089	Ferredoxin protein [*Herbaspirillum seropedicae* SmR1] 80%
12	849	Ferredoxin protein [*Herbaspirillum seropedicae* SmR1] 80%
13	363	Nitrogenase stabilizing protein [*Herbaspirillum seropedicae* SmR1] 50%
14	1131	Homocitrate synthase [*Herbaspirillum seropedicae* SmR1] 63%
15	1587	Transmembrane protein [*Ralstonia eutropha* H16] 59%
16	240	Conserved hypothetical protein [*Methylococcus capsulatus* str. Bath] 55%
17	1659	Nif-specific regulatory protein [*Herbaspirillum seropedicae* SmR1] 61%
18	1872	Fe-S protein assembly chaperone HscA [*Methylovorus sp.* SIP3-4] 69%
19	357	Iron-sulfur cluster insertion protein ErpA [*Nitrosomonas europaea*] 69%
20	1563	FeMo cofactor biosynthesis protein [*Herbaspirillum seropedicae* SmR1] 83%
21	753	Conserved hypothetical protein [*Herbaspirillum seropedicae* SmR1] 60%
22	240	Putative NifZ protein [*Methylococcus capsulatus* str. Bath] 60%
23	1161	Aminotransferase class V [*Beijerinckia indica subsp. indica* ATCC 9039] 58%
24	267	Conserved hypothetical protein [*Rhodopseudomonas palustris* BisB18] 43%
25	1629	Rhodanese domain protein [*Beijerinckia indica subsp. indica* ATCC 9039] 55%
26	414	Oxygen-binding (globin) protein [*Herbaspirillum seropedicae* SmR1] 68%
27	426	Two component regulator protein [*Herbaspirillum seropedicae* SmR1] 60%
28	4557	Methyl-accepting chemotaxis transducer [*Acidovorax delafieldii* 2AN] 51%
29	990	RuBisCO operon transcriptional regulator [*Beggiatoa sp.* PS] 54%
30	1422	RubisCO formI large subunit [*Acidithiomicrobium sp*.] 91%
31	333	RubsiCO small subunit [*Acidithiobacillus ferrivorans* SS3] 70%
32	2382	Carboxysome shell polypeptide [*Halothiobacillus neapolitanus* c2] 46%
33	1497	Carboxysome shell carbonic anhydrase [*Halothiobacillus neapolitanus* c2] 58%

a Nucleotide identity of fosmid DX-1A-14 gene to the gene of the organism to which it is most related.

### Transcriptional unit prediction, cloning, expression and reverse transcription PCR

The program FindTerm (http://linux1.softberry.com/berry.phtml) was used to analyze potential transcription terminators. Locations of significant σ^N^, NifA recognition sites in the upstream of genes and of potential transcriptional terminators are presented in Table.3. Reverse transcription PCR was performed to confirm the co-transcription of *nifQ* and *fixX*. The contiguous *fixX* and *nifQ* gene sequence was amplified from fosmid DX-1A-14 by polymerase chain reaction (PCR), using the oligonucleotides Fix_nif_F 5′-CGCGCTAGCATGAGCGGACTGCGTGTCGAAG-3′ (NheI site is underlined) and Fix_nif_R 5′-CAGGAATTCTCAGGCCGCCCGCAGGGCTTG-3′ (EcoRI site is underlined). The amplified DNA fragment was cleaved with restriction endonuclease NheI and EcoRI, and ligated in the NheI/EcoRI-digested plasmid pET-28a (Novagen, Nadison, WI). Then, the resulting plasmid pET-28NIF containing *fixX* and *nifQ* was transformed into *E. coli* strain BL21. The *E. coli* strain BL21 with plasmid pET-28NIF was grown in Terrific Broth. The growth was achieved aerobically at 37°C in 20 ml of media in a 50-mililiter flask, and cells were induced with 0.1 mM isopropyl β-D-thiogalactoside at A600 ∼0.6. After one hour incubation, cells were harvested by centrifugation at 4°C at 10,000 rpm for 2 min in a 5415R centrifuge (Eppendorf AG, Hamburg, Germany). Total RNA was extracted using Total RNA Isolation Kit (Omega Bio-Tek, Doraville, USA) and then treated with RNase-free DNase I (QIAGEN, Carlsbad, CA) to digest residual chromosomal DNA. Total RNA was quantified at A260 and A280 with NanoDrop® ND-1000 spectrophotometer (NanoDrop Technologies, Wilmington, USA). Reverse transcription PCR was performed using ImProm-II™ Reverse Transcription System, using the above mentioned primers. PCR products were visualized on 2% agarose gels in TAE buffer, purified directly with the QIAquick PCR purification kit (Qiagen, Germany) and sequenced bidirectionally.

**Table 3:** Gene products in the *nif-fix* cluster of fosmid DX-1A-14.

Gene	Product size (kDa)	Function	Regulatory feature	Organisms with closest match^a^	% Identity^b^
*nifK*	22.5	Nitrogenase structure; Fe-Mo protein beta		*H. seropedicae* SmR1	80
*nifE*	55.3	Fe-Mo cofactor synthesis	σ^N^-binding sites	*B. vietnamiensis* G4	79
*nifN*	49.3	Fe-Mo cofactor synthesis		*H. seropedicae* SmR1	68
*nifX*	14.9	Fe-Mo cofactor synthesis		*H. seropedicae* SmR1	74
*nrf1*	17.3	NifX-associated protein		*H. seropedicae* SmR1	71
*orf1*	7.5	Unknown		*B. indica* ATCC 9039	58
*fdxB*	11.5	Ferredoxin		*R.benzoatilyticus* JA2	63
*nifQ*	21.4	Fe-Mo cofactor synthesis	ρ-independent terminator	*C.Accumulibacter*UW-1	52
*fixX*	10.6	Electron transfer; ferredoxin-like protein		*H. seropedicae* SmR1	74
*fixC*	47.8	Electron transfer; electron transfer flavoprotein quinone oxidoreductase		*H. seropedicae* SmR1	79
*fixB*	39.3	Electron transfer; electron transfer flavoprotein alpha subunit		*H. seropedicae* SmR1	80
*fixA*	30.4	Electron transfer; electron transfer flavoprotein beta subunit		*H. seropedicae* SmR1	80
*nifW*	13.5	Protection o f the Fe-Mo protein; Maturation and activation		*H. seropedicae* SmR1	50
*nifV*	40.9	Fe-Mo cofactor synthesis	NifA-,σ^N^-binding sites	*H. seropedicae* SmR1	63
*Aer*	55.9	Aerotaxis sensor receptor	σ^N^-binding sites; ρ-independent terminator	*R. eutropha* H16	59
*orf2*	8.6	Unknown		*M.capsulatus* str. Bath	54
*nifA*	61.2	Transcriptional activator	NifA-,σ^N^-binding sites	*H. seropedicae* SmR1	61
*hscA*	66.4	Fe-S protein assembly; molecular chaperone		*Methylovorus sp.* SIP3	69
*erpA*	12.6	Iron-sulfur cluster insertion	σ^N^-binding sites	*N. europaea*	69
*nifB*	56.9	Fe-Mo cofactor synthesis	NifA-,σ^N^-binding sites	*H. seropedicae* SmR1	82
*orf3*	28.3	Unknown		*H. seropedicae* SmR1	59
*nifZ*	8.4	Activation and Maturation		*M.capsulatus* str. Bath	60
*nifS*	40.2	Cysteine desulfurase; Maturation and activation		*B. indica* ATCC 9039	57
*orf4*	9.6	Unknown		*R. palustris* BisB18	43
*sseA*	59.2	Rhodanese; activation of apoferredoxins		*B. indica* ATCC 9039	54
*nrf2*	15.6	Hemoglobin; oxygen-binding protein		*H. seropedicae* SmR1	68
*nrf3*	15.0	Two component response regulator	σ^N^-binding sites	*H. seropedicae* SmR1	59
*mcpA*	54.4	Chemotaxis		*A. delafieldii* 2AN	48

a Organism in which the gene products most similar to that of the nif-fix cluster was found. Organism: *Herbaspirillum seropedicae, Burkholderia vietnamiensis, Beijerinckia indica, Rubrivivax benzoatilyticus, Candidatus Accumulibacter phosphatis clade IIA, Ralstonia eutropha, Methylococcus capsulatus, Methylovorus sp., Nitrosomonas europaea, Rhodopseudomonas palustris, Acidovorax delafieldii.*

b Identity of the deduced anomic acid sequence of gene product to the gene product of the organism to which it is most related.

### Nucleotide sequence accession numbers

The bioproject of metagenomic sequencing has been registered in NCBI database, and assigned accession number PRJNA202393. And the genomic DNA sequences of fosmid DX-1A-14 and DX-4H-17 containing *nif* gene cluster were submitted to GenBank and have been compiled under accession number JX308284 and JQ815896.

## Results and Discussion

### Detection of *nif* genes in acid mine drainage

**Figure 1:**
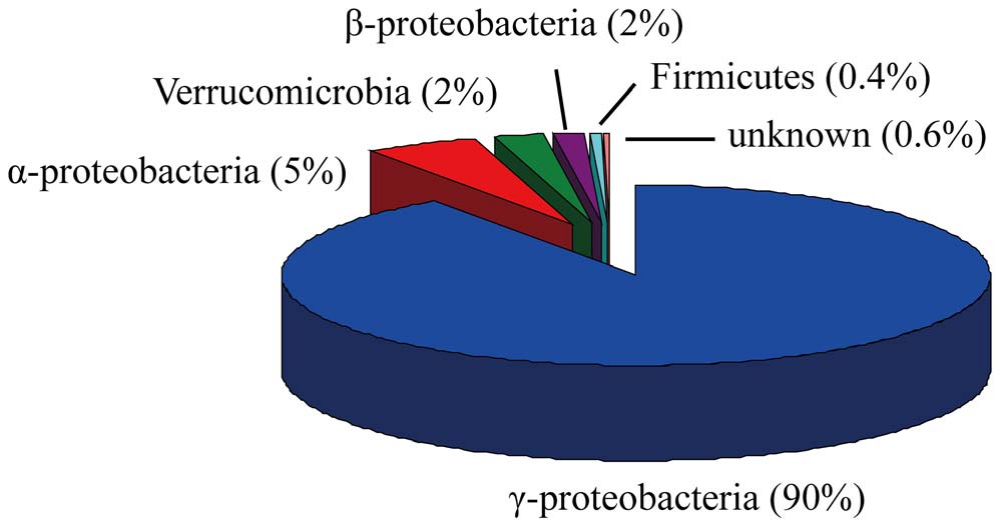
Classification of total *nif* genes obtained from metagenomic sequencing reads. 742 *nif* sequences were classified into different phyla based on the similarities to the known sequences. The community is massively dominated by γ*-proteobacteria*, followed in smaller amounts by *α-, β-proteobacteria* and *Verrucomicrobia*.

To obtain as many *nif* genes as possible in AMD environment, we performed metagenomic sequencing of the AMD sample from Dexing Copper Mine in China. Metagenomic sequencing yielded a total of 640,892 reads and 300 Mb of raw sequence with the average length of 471 bp. One of the important objectives in this metagenomic study is to determine the microbial community structure of nitrogen-fixing microorganisms in the AMD sample. Since *nif* genes are rather conservative and indispensable in nitrogen-fixing microorganisms, they are often used to get reliable phylogenetic affiliation [1]. In our case, 742 sequences were identified as *nif* genes including *nifH*, *nifD*, *nifK* genes and various additional genes from the metagenomic sequencing data (Table. 1). And they were classified into different phyla based on the similarities to the known sequences. The result showed that the community is massively dominated by γ*-proteobacteria* (90%), followed in smaller amounts by *α-, β-proteobacteria* and *Verrucomicrobia* (Fig.1). Furthermore, almost all of *nif* genes classified in γ*-proteobacteria* have their best hits (>90% amino acid identity) with *Acidithiobacillus ferrooxidans* or *Acidithiobacillus ferrivorans*. Thus, it is reasonable that the genus *Acidithiobacillus* greatly contribute to nitrogen fixation in the AMD community. Except the *nif* genes classified in γ*-proteobacteria*, the other *nif* gene sequences are most similar to known microorganisms, ranging from 30% to 85% identity. The discovery of these novel *nif* genes suggested that more diazotrophs survive in the AMD community.

**Figure 2:**
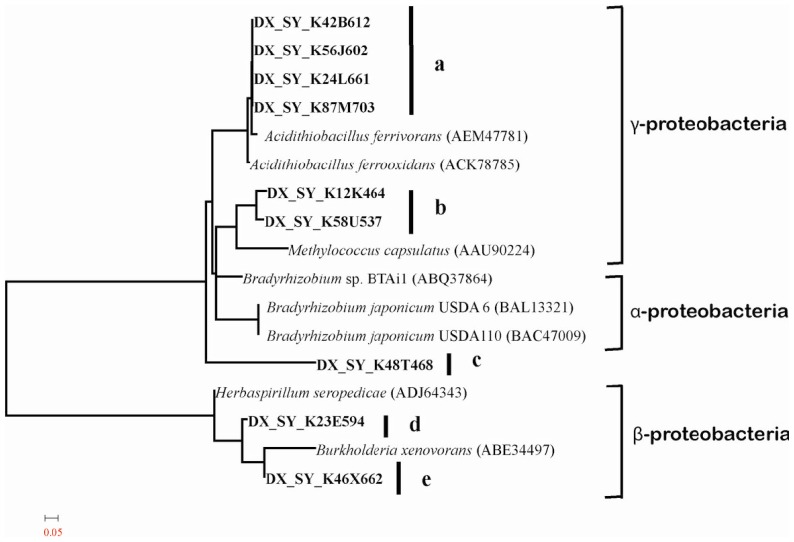
Phylogenetic tree of *nifK* genes. The phylogenetic tree was constructed by the neighbor-joining method using MEGA, version 4.0 with 1000 bootstrap repetitions. The sequences obtained from metagenomic sequencing of acid mine drainage are designated DX_SY_, followed by their number in library. These sequences are shown in bold. Only some representatives of 93 *nifK* sequences are shown here. The scale represents the number of amino acid substitutions per site.

Here, we also used the phylogenetic tree of *nifK* gene to analyze the taxonomic distribution of nitrogen-fixing microorganisms. A total of 93 *nifK* sequences were aligned and fell into five distinct groups, a to e (Fig. 2). The amino acid sequence heterogeneity within each group was moderate, while the differences of amino acid sequences among groups were significant. Group a sequences had best similarities within *Acidithiobacillus* genera, and 92% of *nifK* sequences were classified into this group. This result fitted quite well with the analysis of community structure using *nif* genes. Group b, sharing 68% amino acid similarity with group a, was assigned in *Gammaproteobacteria* group in the *nifK* phylogenetic tree. These *nifK* genes in group b have their best similarities with *Methylococcus capsulatus*. Only one sequence fell into group c, but its taxonomic status can not be determined. And group d and e were assigned into *Betaproteobacteria* group in the *nifK* phylogenetic tree. They have best hits within *Herbaspirillum* and *Burkholderia* genera, separately. These results suggested that the phylogenetic diversity of nitrogen-fixing microorganisms is much higher than previously thought in the AMD community.

### Gene annotation and sequence analysis

After microarray hybridization and sequencing, we found that most of *nifK* genes in positive clones had best hits with *Acidithiobacillus ferrooxidans*, which is consistent with the result of metagenomic sequencing. However, the *nifK* gene of fosmid DX-1A-14 showed novel sequence characteristics. The full-length fosmid sequencing confirmed that the fosmid harbored a novel *nif* gene cluster. Meanwhile, fosmid DX-4H-17 which harbored a large overlap sequence containing no *nifK* gene (approximately 25 kbp) with fosmid DX-1A-14 was chosen by nonspecific binding of *nifK* probe and fully-sequenced. Thus, a 32.5-kb nucleotide sequence with 33 open reading frames (ORFs) was obtained based on the assembly of fosmid DX-1A-14 and DX-4H-17. Gene annotation indicated that most ORFs showed sequence homologies with *nif*, *fix* and associated genes, and 5 ORFs in the end of sequence were identified as RubisCO associated genes. Gene length, Gene annotation, closest strain hits and percent similarity in fosmid DX-1A-14 are summarized in Table.2.

**Figure 3:**
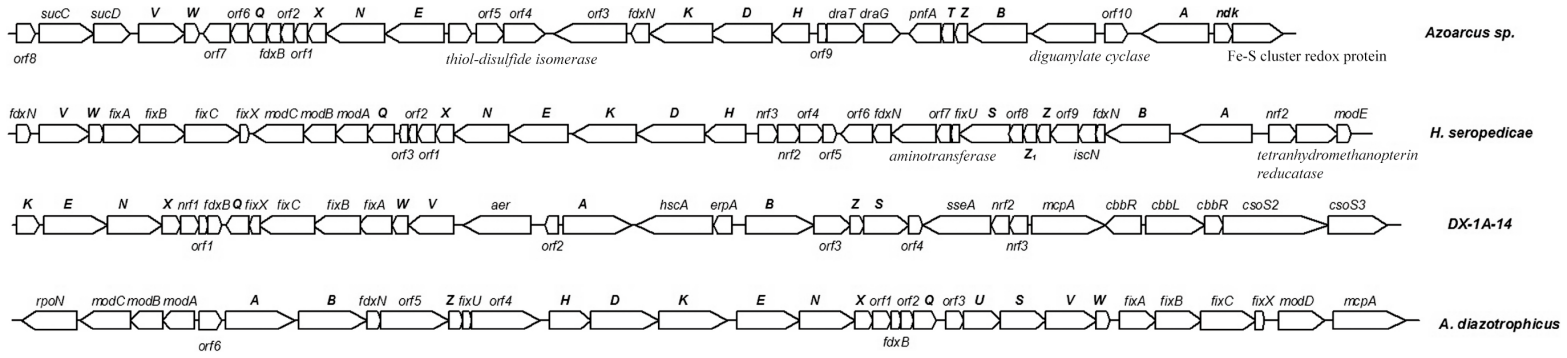
Comparison of the physical organization of *nif*, *fix*, and associated genes from fosmid DX-1A-14 with those from three closest organisms. Organisms: *Azoarcus sp.*, *Herbaspirillum seropedicae*, *Acetobacter diazotrophicus*. The *nif* genes are highlighted in bold. *nrf*, *nif* associated genes; *orf*, hypothetical protein. The structure of *nif* gene cluster differs greatly from those of unknown microorganisms. The *nif* gene cluster does not contain *modABC* genes occurring in *H. seropedicae*, and RubisCO gene cluster can not be identified in the *nif* gene cluster region of *H. seropedicae*. *nifQ* gene is clustered together with *nifENX* and *fdxB* genes in *A. diazotrophicus*, while *nifV*, *nifW*, *fixABCX*, and *nifQ* genes constitute a single operon in fosmid DX-1A-14.

The *nifH*, *nifD*, and *nifK* genes are required for the functional nitrogenase in almost all diazotrophs. Unfortunately, only a truncated *nifK* gene was located in the end of the nucleotide sequence without *nifH* and *nifD* genes. It is highly possible that *nifH*, *nifD* and part of *nifK* sequence were truncated when fosmid library was constructed. Furthermore, iron-sulfur (FeS) cluster is an important component for nitrogenase and both NifS and NifU are required for the formation of iron-sulfur cluster in nitrogenase in *Azotobacter vinelandii* [26]. However, we could identify a *nifS* gene but *nifU* in the nucleotide sequence. It is reported that NifU might act as a scaffold for the assembly of the Fe-S cluster required for the maturation of the nitrogenase complex [27]. Two ORFs encoding Fe-S cluster assembly accessory proteins HscA and ErpA were located between *nifA* and *nifB* (Fig. 3). HscA is reported to be a molecular chaperone of Fe-S cluster assembly in the ISC (iron-sulfur cluster) system [28], and ErpA is a Fe-S cluster insertion protein, which is required for the delivery of Fe-S clusters. Besides, a gene encoding rhodanese was located in the genomic region neighboring the *nifS* gene. There is evidence to suggest that rhodanese can mobilize sulfur from thiosulfate for *in vitro* formation of Fe-S clusters [29]. Besides, these genes are always present in *nif* gene clusters in other diazotrophs [13], [14]. It is possible that these proteins mentioned above were involved in the assembly Fe-S cluster for nitrogenase.

Sequence comparison with known *nif* gene clusters in diazotrophs revealed that many genes in fosmid DX-1A-14 were generally most similar to those found in *β-proteobacteria*, with 15 genes products being most like those of *Herbaspirillum seropedicae*, ranging from 50% (for *nifW*) to 82% (for *nifB*) identity (Table. 2). NifZ was most similar to the gene product of *Methylococcus* species, member of γ-*proteobacteria* (60% identity). And NifE was most similar to gene products of *Burkholderia vietnamiensis* (79% identity). NifS and NifQ had low levels of sequence identity of 57% with *Beijerinckia indica subsp. indica* ATCC 9039 and 52% with *Candidatus Accumulibacter phosphatis clade IIA str.* UW-1, separately. And all *fix* gene products were most similar to those of *H. seropedicae*. It is worth pointing out that all of these species mentioned above were not reported to survive in AMD environment and that gene identities are rather low. This may result from the insufficient knowledge of nitrogen-fixing genes in AMD environment. Besides, the phylogeny of RubisCO gene cluster in fosmid DX-1A-14 is significantly inconsistent with *nif* gene cluster. Most gene products of RubisCO gene cluster were generally most similar to those found in γ*-proteobacteria* (Table.2). Thus, taxonomic status of the nucleotide sequence cannot be assign into known microorganisms.

### Organization of nitrogen-fixing genes

In many genomes of nitrogen-fixing bacteria, *nif* genes are always organized into several clusters. In *Klebsiella pneumoniae*, a total of 20 *nif*-specific genes are organized in a single cluster spanning approximately 24 kbp genomic region [30]. In *Rhizobium meliloti* and *Azotobacter vinelandii*, the *nif* genes are also highly clustered, forming several small *nif* gene clusters in the chromosome [2], [31]. Here, comparative genome analysis with the *nif* gene clusters of closest diazotrophs was performed (Fig.3).

One portion of the *nif-fix* cluster of this nucleotide sequence (*nifK* to *nifW*) is most like that of *H. seropedicae*. In almost all diazotrophs, the structural genes *nifHDK* for the nitrogenase complex were distinctly clustered. Transcriptional analysis determined that the whole *nifHDKENXorf1orf2orf3* operon is transcribed from a single promoter located upstream of the *nifH* gene in *H. seropedicae* [32]. In fosmid DX-1A-14, a high GC sequence was present, which exhibited strong homology to the *nif* promoter consensus sequence (GG-N10-GC) [33], in intergenic spacing between *nifK* and *nifE* gene, suggesting that the clustered genes starting from *nifE* may form a new operon. Another primary divergence in gene organization of this nucleotide sequence and *H. seropedicae* is that the *nifQ* and *modABC* genes formed a cluster in the downstream from *nifHDKENXorf 1 orf 2 orf 3* operon and constitute a single operon in *H. seropedicae* [34], but *modABC* genes were not found in this nucleotide sequence (Fig. 3). The *modABC* genes encode a high-affinity molybdate transport system in *H. seropedicae* [35]. Another organization of *nifQ* gene that is different from that of *H. seropedicae* is found in *A. diazotrophicus* (Fig.3). *nifQ* gene was clustered together with *nifENX* and *fdxB* genes in the *nif-fix* cluster of *A. diazotrophicus* [7]. Unexpectedly, the *nifQ* gene in this nucleotide sequence had a divergent orientation with *nifENX* and *fdxB* genes, although *nifQ* is also located in the immediate downstream of *fdxB* gene (Table.2). Further sequence analysis suggested that *nifV*, *nifW*, *fixABCX*, and *nifQ* genes that had continuous arrangement in this nucleotide sequence were organized in a single gene cluster without intergenic spacing (Fig.3).

On the other hand, many additional *nif* genes including *nifA*, *nifB*, *nifS*, and *nifZ* interspersed in the upstream of *nifHDK* in *A. diazotrophicus* and *H. seropedicae*, however, more additional *nif* genes were located in the downstream of *nifHDK* in fosmid DX-1A-14 (Fig.3). And *nifA* gene in this nucleotide sequence is not linked to the other *nif* genes like that in *A. diazotrophicus*. The majority of the *nif* gene promoters is of σ^N^-dependent type and activated by NifA protein [36]. Thus, *nifA* gene is always considered to be the most important regulatory gene in nitrogen fixation process. In many nitrogen-fixing bacteria, *nifB* forms a *nif* gene cluster with other genes involved in Fe-Mo cofactor synthesis such as *nifQ*, *nifW*, and *fdxB* genes [37], but these genes are not adjacent to the *nifB* gene in fosmid DX-1A-14. In fact, the *nifB*, *orf3*, *nifZ, nifS*, and *orf 4* genes was organized in a single gene cluster without intergenic spacing in fosmid DX-1A-14. However, *nifS* genes were always clustered with *nifU*, *nifV*, *nifW* in most nitrogen-fixing bacteria [26].

Except these *nif-fix* genes, another important feature of the *nif-fix* cluster was that some other genes interspersed in the fragment region of fosmid DX-1A-14. For example, two chemotaxis genes, *Aer* and *mcpA*, were found in fosmid DX-1A-14. These chemotaxis genes may be responsible for chemotactic responses to oxygen levels or nutrition [38]. What's interesting is that a truncated RubisCO gene cluster including *cbbR*, *cbbL*, *cbbR*, *csoS2*, and *csoS3* genes is located in the immediate downstream of *nif-fix* cluster (Table. 2). The analysis of fosmid DX-4H-17 revealed that a complete RubisCO gene cluster occurs in the organism to which the fosmid DX-1A-14 sequence belongs.

### Transcriptional organizations of the *nif-fix* cluster

**Figure 4:**
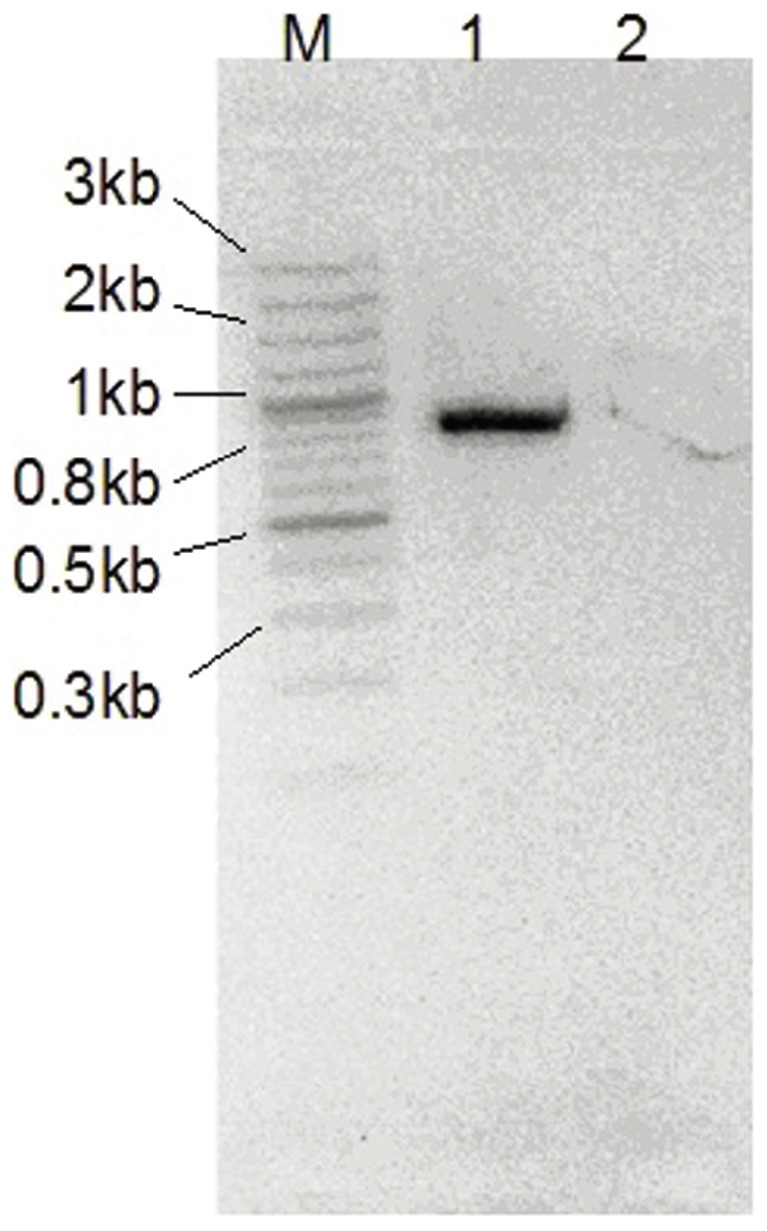
Reverse transcription PCR of *fixX* and *nifQ* gene fragment. Lane M, 100; lane 1, PCR products with the template of total RNA extraction; lane 2, PCR products with the template of total RNA extracting digested by RNase.

The transcriptional organizations were predicted by sequence analysis, which revealed NifA-, σ^N^-binding sites upstream of operons required, respectively, for *nif* gene transcriptional activation and for *nif* promoter recognition in all proteobacterial diazotrophs [35]. The transcriptional organizations of the *nif-fix* gene cluster in fosmid DX-1A-14 showed several interesting features (Table.3). The cotranscription of *nifENX nrf1 orf1 fdxB* was identified by a typical σ^N^-binding site (GG-N10-CG) in the upstream of *nifE*. The continuous arrangement and suitable intergenic spacing from *nifHDK* genes also suggested the six genes possibly compose an operon. However, there was no consensus sequences (TGT-N10-ACA) [33] for the typical NifA binding sites in the promoter region. But in the upstream of the *nifV* gene, two typical NifA binding site consensus sequences were detected from position -216 to -200: TGTATCAAACCATACA and -126 to -110: TTTACGAAGGAAAACA. This finding suggested that there could be an independent transcriptional unit starting from the *nifV* gene in fosmid DX-1A-14. The continuous arrangement and unusual overlaps among the 3′ and 5′ ends of adjacent genes indicated the cotranscription of *nifV* to *nifQ*. Reverse transcription PCR also confirmed that the *nifQ* gene fell into the same transcription unit with *fixX* gene (Fig.4). This has not been reported in other diazotrophs before. Locations of possibly significant NifA, σ^N^ recognition sites upstream of genes and potential transcription terminators downstream of genes were also presented in Table.3.

In conclusion, we isolated 742 *nif* genes using metagenomic sequencing from AMD community. Metagenomic analysis suggested that the AMD community is massively dominated by the genus *Acidithiobacillus*, but the phylogenetic diversity of nitrogen-fixing microorganisms is much higher than previously thought in the AMD community. To understand the structure of *nif* gene clusters of uncultured microorganisms in AMD community, a metagenome microarray was constructed to screen novel *nif* gene clusters. A 32.5-kb genomic sequence harboring *nif-fix* gene cluster was isolated from the metagenome of AMD community. Most of *nif*, *fix* genes were individually similar to those of *H. seropedicae*, but the organization of the *nif-fix* cluster of this nucleotide sequence showed some distinct features. The NifA-, σ^N^-binding sites in the promoter region indicated that distinct transcription units of *nif* genes exist in the gene cluster. These results provided a sketch about the structure of nitrogen-fixing gene cluster in the uncultured microorganisms of AMD community.

## References

[1] Young J (1992) Phylogenetic classification of nitrogen-fixing organisms. Biological nitrogen fixation 23: 43–86.

[2] Jacobson M, Brigle K, Bennett L, Setterquist R, Wilson M, et al (1989) Physical and genetic map of the major *nif* gene cluster from *Azotobacter vinelandii* . Journal of Bacteriology 171: 1017–1027. 2644218 10.1128/jb.171.2.1017-1027.1989 PMC209696

[3] Pedrosa F, Teixeira KRS, Machado I, Steffens M, Klassen G, et al (1997) Structural organization and regulation of the nif genes of *Herbaspirillum seropedicae* . Soil Biology and Biochemistry 29: 843–846.

[4] Yan Y, Yang J, Dou Y, Chen M, Ping S, et al (2008) Nitrogen fixation island and rhizosphere competence traits in the genome of root-associated *Pseudomonas stutzeri* A1501. Proceedings of the National Academy of Sciences 105: 7564. 10.1073/pnas.0801093105 PMC2396677 18495935

[5] Adams TH, McClung CR, Chelm BK (1984) Physical organization of the *Bradyrhizobium japonicum* nitrogenase gene region. Journal of Bacteriology 159: 857–862. 6090394 10.1128/jb.159.3.857-862.1984 PMC215737

[6] Masepohl B, Drepper T, Paschen A, Gross S, Pawlowski A, et al (2002) Regulation of nitrogen fixation in the phototrophic purple bacterium *Rhodobacter capsulatus* . Journal of molecular microbiology and biotechnology 4: 243–248. 11931554

[7] Lee S, Reth A, Meletzus D, Sevilla M, Kennedy C (2000) Characterization of a major cluster of *nif*, *fix*, and associated genes in a sugarcane endophyte, *Acetobacter diazotrophicus* . Journal of Bacteriology 182: 7088–7091. 11092875 10.1128/jb.182.24.7088-7091.2000 PMC94840

[8] Kallas T, Coursin T, Rippka R (1985) Different organization of *nif* genes in nonheterocystous and heterocystous *cyanobacteria* . Plant molecular biology 5: 321–329. 24306924 10.1007/BF00020630

[9] Earl C, Ronson C, Ausubel F (1987) Genetic and structural analysis of the Rhizobium meliloti *fixA*, *fixB*, *fixC*, and *fixX* genes. Journal of Bacteriology 169: 1127–1136. 3029021 10.1128/jb.169.3.1127-1136.1987 PMC211910

[10] Edgren T, Nordlund S (2004) The *fixABCX* genes in *Rhodospirillum rubrum* encode a putative membrane complex participating in electron transfer to nitrogenase. Journal of Bacteriology 186: 2052–2060. 15028689 10.1128/JB.186.7.2052-2060.2004 PMC374401

[11] Baker BJ, Banfield JF (2003) Microbial communities in acid mine drainage. FEMS Microbiology Ecology 44: 139–152. 19719632 10.1016/S0168-6496(03)00028-X

[12] Bond PL, Smriga SP, Banfield JF (2000) Phylogeny of microorganisms populating a thick, subaerial, predominantly lithotrophic biofilm at an extreme acid mine drainage site. Applied and Environmental Microbiology 66: 3842–3849. 10966399 10.1128/aem.66.9.3842-3849.2000 PMC92229

[13] Levicán G, Ugalde JA, Ehrenfeld N, Maass A, Parada P (2008) Comparative genomic analysis of carbon and nitrogen assimilation mechanisms in three indigenous bioleaching bacteria: predictions and validations. BMC genomics 9: 581. 19055775 10.1186/1471-2164-9-581 PMC2607301

[14] Cárdenas JP, Valdés J, Quatrini R, Duarte F, Holmes DS (2010) Lessons from the genomes of extremely acidophilic bacteria and archaea with special emphasis on bioleaching microorganisms. Applied microbiology and biotechnology 88: 605–620. 20697707 10.1007/s00253-010-2795-9

[15] Handelsman J (2004) Metagenomics: application of genomics to uncultured microorganisms. Microbiology and Molecular Biology Reviews 68: 669–685. 15590779 10.1128/MMBR.68.4.669-685.2004 PMC539003

[16] Zhou J, Bruns MA, Tiedje JM (1996) DNA recovery from soils of diverse composition. Applied and Environmental Microbiology 62: 316–322. 8593035 10.1128/aem.62.2.316-322.1996 PMC167800

[17] Guo X, Yin H, Cong J, Dai Z, Liang Y, et al (2013) RubisCO Gene Clusters Found in a Metagenome Microarray from Acid Mine Drainage. Applied and Environmental Microbiology 79: 2019–2026. 23335778 10.1128/AEM.03400-12 PMC3592212

[18] Rhee S-K, Liu X, Wu L, Chong SC, Wan X, et al (2004) Detection of genes involved in biodegradation and biotransformation in microbial communities by using 50-mer oligonucleotide microarrays. Applied and Environmental Microbiology 70: 4303–4317. 15240314 10.1128/AEM.70.7.4303-4317.2004 PMC444823

[19] Chaisson MJ, Pevzner PA (2008) Short read fragment assembly of bacterial genomes. Genome research 18: 324–330. 18083777 10.1101/gr.7088808 PMC2203630

[20] Delcher AL, Harmon D, Kasif S, White O, Salzberg SL (1999) Improved microbial gene identification with GLIMMER. Nucleic acids research 27: 4636–4641. 10556321 10.1093/nar/27.23.4636 PMC148753

[21] Aziz R, Bartels D, Best A, DeJongh M, Disz T, et al (2008) The RAST Server: rapid annotations using subsystems technology. BMC genomics 9: 75. 18261238 10.1186/1471-2164-9-75 PMC2265698

[22] Altschul SF, Madden TL, Schäffer AA, Zhang J, Zhang Z, et al (1997) Gapped BLAST and PSI-BLAST: a new generation of protein database search programs. Nucleic acids research 25: 3389–3402. 9254694 10.1093/nar/25.17.3389 PMC146917

[23] Eddy SR (2008) A probabilistic model of local sequence alignment that simplifies statistical significance estimation. PLoS computational biology 4: e1000069. 18516236 10.1371/journal.pcbi.1000069 PMC2396288

[24] Carver TJ, Rutherford KM, Berriman M, Rajandream MA, Barrell BG, et al (2005) ACT: the Artemis comparison tool. Bioinformatics 21: 3422–3423. 15976072 10.1093/bioinformatics/bti553

[25] Tamura K, Dudley J, Nei M, Kumar S (2007) MEGA4: molecular evolutionary genetics analysis (MEGA) software version 4.0. Molecular biology and evolution 24: 1596–1599. 17488738 10.1093/molbev/msm092

[26] Jacobson MR, Cash VL, Weiss MC, Laird NF, Newton WE, et al (1989) Biochemical and genetic analysis of the *nifUSVWZM* cluster from *Azotobacter vinelandii* . Molecular and General Genetics MGG 219: 49–57. 2615765 10.1007/BF00261156

[27] Agar J, Yuvaniyama P, Jack R, Cash V, Smith A, et al (2000) Modular organization and identification of a mononuclear iron-binding site within the NifU protein. Journal of Biological Inorganic Chemistry 5: 167–177. 10819462 10.1007/s007750050361

[28] Johnson DC, Dean DR, Smith AD, Johnson MK (2005) Structure, function, and formation of biological iron-sulfur clusters. Annu Rev Biochem 74: 247–281. 15952888 10.1146/annurev.biochem.74.082803.133518

[29] Jutabha P (2001) Biochemical and genetic characterization of mercaptopyruvate sulfurtransferase and paralogous putative sulfurtransferases of *Escherichia coli*: Virginia Polytechnic Institute and State University.

[30] Arnold W, Rump A, Klipp W, Priefer UB, Pühler A (1988) Nucleotide sequence of a 24,206-base-pair DNA fragment carrying the entire nitrogen fixation gene cluster of *Klebsiella pneumoniae* . Journal of molecular biology 203: 715–738. 3062178 10.1016/0022-2836(88)90205-7

[31] Corbin D, Ditta G, Helinski D (1982) Clustering of nitrogen fixation (*nif*) genes in *Rhizobium meliloti* . Journal of Bacteriology 149: 221–228. 6274844 10.1128/jb.149.1.221-228.1982 PMC216613

[32] Machado I, Yates M, Machado H, Souza E, Pedrosa F (1996) Cloning and sequencing of the nitrogenase structural genes *nifHDK* of *Herbaspirillum seropedicae* . Brazilian journal of medical and biological research = Revista brasileira de pesquisas medicas e biologicas/Sociedade Brasileira de Biofisica[et al] 29: 1599. 9222418

[33] Pawlowski K, Klosse U, De Bruijn F (1991) Characterization of a novel *Azorhizobium caulinodans* ORS571 two-component regulatory system, NtrY/NtrX, involved in nitrogen fixation and metabolism. Molecular and General Genetics MGG 231: 124–138. 1661370 10.1007/BF00293830

[34] Pedrosa FO, Monteiro RA, Wassem R, Cruz LM, Ayub RA, et al (2011) Genome of *Herbaspirillum seropedicae* strain SmR1, a specialized diazotrophic endophyte of tropical grasses. PLoS genetics 7: e1002064. 21589895 10.1371/journal.pgen.1002064 PMC3093359

[35] Pedrosa F, Benelli E, Yates M, Wassem R, Monteiro R, et al (2001) Recent developments in the structural organization and regulation of nitrogen fixation genes in *Herbaspirillum seropedicae* . Journal of biotechnology 91: 189–195. 11566390 10.1016/s0168-1656(01)00343-1

[36] Fischer HM (1994) Genetic regulation of nitrogen fixation in *rhizobia* . Microbiological reviews 58: 352. 7968919 10.1128/mr.58.3.352-386.1994 PMC372973

[37] Liang Y, Kaminski P, Elmerich C (1991) Identification of a *nifA*-like regulatory gene of *Azospirillum brasilense* Sp7 expressed under conditions of nitrogen fixation and in the presence of air and ammonia. Molecular Microbiology 5: 2735–2744. 1779763 10.1111/j.1365-2958.1991.tb01982.x

[38] Ferrández A, Hawkins AC, Summerfield DT, Harwood CS (2002) Cluster II che genes from *Pseudomonas aeruginosa* are required for an optimal chemotactic response. Journal of Bacteriology 184: 4374–4383. 12142407 10.1128/JB.184.16.4374-4383.2002 PMC135244

